# Outcomes in patients with multiple sclerosis and solid organ cancers treated with immune checkpoint inhibitors

**DOI:** 10.1093/noajnl/vdaf048

**Published:** 2025-03-04

**Authors:** Chanawee Hirunpattarasilp, Pingfu Fu, Seunghee Park Margevicius, Matthew Mirsky, Debora Bruno, Ankit Mangla, Richard Stephen Hoehn, Luke Daniel Rothermel, Iris Yeong-Fung Sheng

**Affiliations:** Princess Srisavangavadhana Faculty of Medicine, Chulabhorn Royal Academy, Bangkok, Thailand; Department of Neurology, University Hospitals Cleveland Medical Center, Cleveland, Ohio, United States; Department of Population and Quantitative Health Sciences, School of Medicine, Case Western Reserve University, Cleveland, Ohio, United States; Department of Population and Quantitative Health Sciences, School of Medicine, Case Western Reserve University, Cleveland, Ohio, United States; Division of Hematology and Oncology, University Hospitals Seidman Cancer Center, Cleveland, Ohio, United States; Department of Medical Oncology, City of Hope Atlanta, Newnan, Georgia, United States; Division of Hematology and Oncology, University Hospitals Seidman Cancer Center, Cleveland, Ohio, United States; Department of Surgery, Division of Surgical Oncology, University Hospitals Cleveland Medical Center, Cleveland, Ohio, United States; Department of Surgery, Division of Surgical Oncology, University Hospitals Cleveland Medical Center, Cleveland, Ohio, United States; Division of Hematology and Oncology, University Hospitals Seidman Cancer Center, Cleveland, Ohio, United States

**Keywords:** immune checkpoint inhibitors, irAEs, multiple sclerosis, retrospective studies

## Abstract

**Background:**

Multiple sclerosis (MS) patients are excluded from studies of immune checkpoint inhibitors (ICIs) due to concern for MS flare. This study aims to report the incidence of MS relapse, immune-related adverse events (irAEs), and cancer outcomes in MS patients with solid-organ malignancy treated with ICIs.

**Methods:**

In this retrospective study, MS patients who received ICIs at University Hospitals Seidman Cancer Center were identified electronically. Information on MS relapses, irAEs, and cancer outcomes after ICI initiation was collected and analyzed.

**Results:**

Twelve patients were included in the study, all of whom had inactive MS. No patient experienced MS relapse or new lesions on brain MRI after ICI initiation. Two patients (16.7%) had severe irAEs (grade ≥ 3), which was acute pneumonitis. No deaths were associated. The objective response rate was 50%. An additional year of age was associated with a 14.2% decrease in the risk of developing severe irAEs (hazard ratio (HR) = 0.858; 95% confidence interval (CI): 0.798–0.922; *P* < .0001) and a 10.3% decrease in the risk of disease progression (HR = 0.897; 95% CI: 0.823–0.976; *P* = .0120). No significant difference in risk of having severe irAEs, overall survival, or progression-free survival was found between MS patients with and without DMTs.

**Conclusions:**

Our study suggests that ICIs could be safely used in patients with inactive MS with a low risk of MS relapse and comparable irAE risk with the general population. Although larger studies are needed for confirmation, the benefit of using ICIs to treat solid-organ malignancies might outweigh the risk of withholding treatment for this population.

**Key Points**

- Patients with inactive MS and cancer treated with ICI had a low risk of MS relapse and experienced irAEs comparable to that of the general population.
- Cancer patients with inactive MS, should not be excluded from ICI treatment consideration.

**Importance of the Study**

This study highlights the safety and efficacy of immune checkpoint inhibitors (ICIs) in cancer patients with multiple sclerosis (MS), who were excluded from most studies of ICIs due to fear of autoimmunity. In our cohort of 12 MS patients with solid-organ cancer with inactive MS, we found no MS relapse and a low irAE risk comparable to those previously reported in the general population. We also showed that ICIs could be effective for MS patients with cancer, regardless of concurrent MS disease-modifying therapy (DMTs). These findings have implications for patient care by providing the possibility of using ICIs for inactive MS patients with cancer and showing that continuation of DMTs might be reasonable. The safety and efficacy profile could also be the basis for larger studies and trials for patients with autoimmune conditions.

Cancer treatment was revolutionized by the introduction of immune checkpoint inhibitors (ICIs) in 2011.^1,2^ These humanized monoclonal antibodies block molecules that regulate T-cell function such as cytotoxic T-lymphocyte antigen 4 (CTLA-4) and programmed cell death 1 (PD-1) or their ligands,^2^ thereby promoting T-cell response to eradicate cancer cells. ICIs (eg pembrolizumab, atezolizumab, and durvalumab) have improved overall survival (OS) and produced durable responses in various cancer types, including melanoma, lung cancers, and breast cancers.^2–4^ However, disinhibited immune responses can lead to autoimmunity and immune-related adverse effects (irAEs).^2,5^ Complications in the nervous system can occur in up to 4% of the patients,^5,6^ including *de novo* and exacerbation of multiple sclerosis (MS), an autoimmune disease mediated by autoreactive T cells causing inflammatory demyelination in the central nervous system.^6,7^

With the concern for autoimmunity from using ICIs, most clinical trials excluded patients with autoimmune diseases.^1,4,8^ As a result, there is an opportunity loss for cancer treatment given the lack of data on the efficacy and safety of ICIs for this population. This is particularly true for patients with MS. MS is not only the most common cause of nontraumatic disability in young adults with a prevalence of 2.8 million people worldwide,^9^ but also has been associated with an increased risk of cancer compared to controls.^10^

Three case reports have shown devastating MS exacerbation with the use of ICIs.^11–13^ Newer small descriptive studies showed that MS patients tolerated the ICIs well, with a reported MS relapse rate of 0–17%^14–18^; attributing the MS relapse, at least in part, to discontinuation of MS disease-modifying therapies (DMTs).^16,17^ A recent study with 66 MS patients showed a relapse rate of 3%^19^ and a meta-analysis found a pooled relapse rate of 5.45 per 100 patient-year.^20^ However, data on cancer outcomes and influencing factors of safety and efficacy of ICI use in this population were limited in these studies.

In this article, we explore the safety and efficacy of ICIs in MS patients with solid tumors. The primary objective is to determine the rate of MS relapse, and the secondary objectives are to report the rate of other irAEs and cancer outcomes in this population, which has not been shown in prior studies. An additional objective is to identify other factors that might affect the safety and efficacy of ICIs in this population (such as sex, race, Charlson Comorbidity Index, smoking, and concurrent MS DMTs).

## Materials and Methods

### Study Design

We conducted a retrospective descriptive study by electronically identifying patients with preexisting MS who underwent ICI treatment for solid organ cancers at University Hospitals Seidman Cancer Center from inception to May 2024. Inclusion criteria were (1) adults of more than 18 years of age, (2) diagnosis of MS preceding ICI initiation, and (3) receipt of ICI treatment for at least 1 cycle for solid organ tumors.

### Clinical Variables and Outcomes

We collected clinical variables, including patient characteristics, MS disease and current DMTs, and cancer diagnosis and treatment. The primary outcome was the incidence rate of MS relapse (defined as new neurological symptoms or worsening of old symptoms in the absence of infection or change in temperature, lasting for 24 h or more) and/or new active T2 or gadolinium-enhancing lesions on brain magnetic resonance imaging (MRI) after ICI initiation. Other outcomes of interest were incidence rates of other irAEs and cancer outcomes. We used revised Response Evaluation Criteria in Solid Tumors (RECIST) to evaluate tumor response.^21^ The data were anonymized and managed using the Research Electronic Data Capture (REDCap) application.

### Protocol Registrations and Approval

The study was approved by the institutional review board of University Hospitals Cleveland Medical Center.

### Statistical Analysis

Incidence rates were calculated as the sum of all cases with events of interest divided by the population size. Time to an immune-related adverse event (TTAE) was measured from the onset of immune therapy to the date of having irAE and was censored at the date of last follow-up for those without irAE with death as a competing risk. The objective response rate was defined as the percentage of patients acquiring a complete or partial response to ICIs. Durable response rate was calculated as the percentage of patients who had a complete or partial response to ICIs lasting at least 6 months. The overall survival (OS) was measured from the onset of immunotherapy to the date of death and was censored at the last follow-up for survivors. The progression-free survival (PFS) was measured from the onset of immunotherapy to the date of relapse or death, whichever occurred earlier, and was censored at the date of the last follow-up for those alive without disease progression.

The cumulative incidence of having irAE (focusing on irAE grade ≥ 3) was estimated, with death as a competing risk. The Fine and Gray method was to compare cumulative incidence between groups.^22^ Survivor distributions were estimated using the Kaplan–Meier methods,^23^ and the differences in OS and PFS between groups were examined by the Wilcoxon test. The effects of demographic or clinical factors on OS and PFS were evaluated using the univariate Cox model.^24^ Due to a small sample size, the effects of important factors on TTAE, OS, and PFS were evaluated using the univariate analysis only. All tests are 2-sided, and *P*-values < .05 were considered statistically significant.

## Results

### Patient Demographics

We identified 12 MS patients (9 female and 3 male) who received ICIs for cancer treatment (Table 1). The median age was 62.2 (range 35.9–77.9) years. Most patients were white (83.3%) and were former smokers (50%).

**Table 1.** Patient Demographics and Characteristics

Factor	Median (range) /frequency (%)
Patient demographics	
Age (y)	62.2 (35.9, 77.9)
Sex Female Male	9 (75.0) 3 (25.0)
Race Black/AA White	2 (16.7) 10 (83.3)
Smoking Current Former Never	3 (25.0) 6 (50.0) 3 (25.0)
Pack years (in current/former) *1 Missing*	45 (26, 150)
MS characteristics	
MS type RRMS Unknown	11 (91.7) 1 (8.3)
MS duration at ICI initiation (y)	22.3 (3.0, 35.5)
MS Treatment at ICI initiation No Yes Dimethyl fumarate (Tecfidera) Glatiramer acetate (Copaxone) Interferon beta-1a (Avonex)	8 (66.7) 4 (33.3) 2 (16.7) 1 (8.3) 1 (8.3)
Cancer characteristics and ICI treatment	
Diagnosis Breast adenocarcinoma Esophageal carcinoma Melanoma Non–small cell lung cancer Small cell lung cancer	2 (16.7) 1 (8.3) 1 (8.3) 6 (50.0) 2 (16.7)
Stage IIA/IIB/III/IIIB IV/IVA/IVB	5 (41.7) 7 (58.3)
ICI given Atezolizumab Durvalumab Pembrolizumab	2 (16.7) 2 (16.7) 8 (66.7)
Number of cycles ICI given	7 (1, 17)
ICI given as an adjuvant No Yes	9 (75.0) 3 (25.0)
Provider Academic Community	11 (91.7) 1 (8.3)
ECOG performance status at ICI initiation 0 1 2	3 (25.0) 3 (25.0) 6 (50.0)
Charlson comorbidity index	8.5 (2.0, 14.0)

Abbreviations: AA, African American; ECOG, Eastern Cooperative Oncology Group; ICI, immune checkpoint inhibitor; MS, multiple sclerosis; RRMS, relapsing-remitting multiple sclerosis.

### MS Characteristics

Almost all patients had relapsing-remitting MS (RRMS; 91.7%) except for 1 patient whose MS type has not been documented (8.3%; Table 1). The median MS duration was 22.3 (range 3.0–35.5) years before ICI treatment. The median time from the last relapse to ICI treatment was 21.3 (range 3.0–35.5) years. Four patients (33.3%) were on DMTs for MS at the time of ICI initiation, specifically dimethyl fumarate (Tecfidera; 16.7%), glatiramer acetate (Copaxone; 8.3%), and interferon beta-1a (Avonex; 8.3%). None of the patients had active MS lesions on initiation of ICI and none had their DMTs discontinued because of the ICI initiation.

### Cancer Characteristics and ICI Treatment

Half of the patients had non–small cell lung cancer (NSCLC; stage IIB, III, and IV; 50%; Table 1). Other cancer diagnoses included breast adenocarcinoma (stage IIA and IV; 16.7%), small cell lung cancer (stage IV; 16.7%), esophageal carcinoma (stage IV; 8.3%), and melanoma (stage IIIB; 8.3%). The majority of the patients received pembrolizumab (66.7%), while the rest were treated with atezolizumab (16.7%) or durvalumab (16.7%). Thus, 66.7% of the patients received PD-1 inhibitors and 33.3% received PDL-1 inhibitors. The median number of cycles of ICIs administered was 7 (1, 17). The ICIs were given as adjuvant therapy in 3 patients (25.0%).

### Outcomes

#### General Outcomes.

—The median and range of follow-up time from initiation of ICI to last follow-up was 14.7 (0.6, 40.2) months. Overall, 2 patients died (16.7%; Table 2). The first patient was a 60-year-old white male with RRMS on glatiramer acetate (Copaxone) and stage IVB NSCLC, who developed acute pneumonitis and passed away due to acute pulmonary embolism in the same admission. The second patient was a 62-year-old white female with small cell lung cancer stage IVB treated with 11 cycles of Atezolizumab and RRMS without concurrent DMTs. The cause of death for the second patient was not documented. Four patients (33.3%) were lost to follow-up or transitioned to hospice care.

**Table 2.** Outcomes after ICI Treatment

Outcome	Median (range) /frequency (%)
General outcomes	
Alive No Lost follow-up/ hospice care Yes	2 (16.7) 4 (33.3) 6 (50.0)
MS outcomes	
Relapses	0 (0)
New brain MRI lesions	0 (0)
irAE outcomes	
irAE No Yes Grade I-II: dermatitis Grade III-IV: pneumonitis	8 (66.7) 4 (33.3) 2 (16.7) 2 (16.7)
Cancer outcomes	
Initial response to ICI CR PR SD PD	1 (8.3) 5 (41.7) 4 (33.3) 2 (16.7)

Abbreviations: CR, complete response; ICI, immune checkpoint inhibitor; irAE, Immune-related adverse event; MRI, magnetic resonance imaging; MS, multiple sclerosis; PD, progressive disease; PR, partial response; SD, stable disease.

#### MS Outcomes.

—None of the included patients had MS relapse after ICI initiation (Table 2). In addition, no patient developed new MRI lesions in the brain after ICI treatment. Hence, the relapse rate was zero for the included population.

#### irAE Outcomes.

—Four patients (33.3%) developed irAEs. Two patients (16.7%) developed severe irAE (irAE grade ≥ 3; Table 2). Both patients developed acute pneumonitis after ICI initiation. The first patient was the 60-year-old NSCLC staged IVB patient with RRMS on Copaxone (mentioned above in general outcomes). The patient developed acute pneumonitis after the first cycle of Pembrolizumab (17 days from ICI initiation) and was treated with dexamethasone until he passed away from acute pulmonary embolism. The second patient was a 37-year-old black female with RRMS (not on DMT) and stage IV breast adenocarcinoma, who developed acute pneumonitis after the second cycle of atezolizumab (69 days from ICI initiation) and transitioned to hospice care after being treated with dexamethasone for irAE.

The cumulative rate of having severe irAE at 2 months was 18% (Supplementary Figure 1). There was no significant difference between MS patients with or without DMTs (*P* = .3908; Supplementary Figure 2).

Univariate analysis with Cox regression on TTAE with death as a competing risk factor (Fine and Gray method) revealed that for every additional year of age, the risk of irAE decreased by 14.2% (HR = 0.858, 95% CI: 0.798–0.922, *P* < .0001; Table 3). There was no significant difference for other factors such as sex, race, Charlson Comorbidity Index, smoking, and concurrent MS DMTs.

**Table 3.** The Results from Cox Regression on Time to an Immune-Related Adverse Event (TTAE; Focusing on irAE grade ≥ 3) with Death as a Competing Risk

Factor	Univariate analysis	
	HR (95% CI)	*P*-value
Age (per 1-y increase)	**0.858 (0.798, 0.922)**	**<.0001**
Sex Female vs. male	0.289 (0.022, 3.715)	.3405
Race Black/AA vs. White	4.472 (0.455, 43.926)	.1988
Charlson Comorbidity Index (per 1-unit increase)	0.876 (0.687, 1.117)	.2862
Smoking Never vs. current/former	2.646 (0.242, 28.976)	.4256
MS treatment (at ICI initiation) No vs. yes	0.354 (0.033, 3.799)	.3908

Abbreviations: AA, African American; CI, confidence interval; HR, hazard ratio; ICI, immune checkpoint inhibitor; MS, multiple sclerosis.

Of note, there were 2 patients (16.7%) with grade I–II irAEs, both of which were dermatitis. The first patient is a 65-year-old white female with RRMS without DMT, who developed recurrent dermatitis after the second cycle of pembrolizumab (43 days from ICI initiation for stage IVA NSCLC and was treated with methylprednisolone, prednisone taper, and triamcinolone cream). The second patient was a 47-year-old white female with RRMS on dimethyl fumarate (Tecfidera) who had dermatitis after the first cycle of pembrolizumab (14 days from ICI initiation) for stage IIA breast adenocarcinoma and was closely monitored. For both patients, ICIs were resumed without any dose adjustment or delay. The first patient had episodes of irAE dermatitis recurrence, which was controlled with oral prednisone daily.

#### Cancer Outcomes.

—The initial response to ICI after a median of 7 cycles included complete response (CR) for 1 patient (8.3%), partial response (PR) for 5 patients (41.7%), stable disease (SD) for 4 patients (33.3%), and progressive disease for 2 patients (16.7%; Table 2). The objective response rate was 50%. Out of 12 patients, 4 patients achieved CR or PR for more than 6 months, thus the durable response rate was 33.3%. For 9 patients whose ICIs were discontinued, median treatment-free survival was 3.8 (0.6, 38.5) months, the other 3 patients were undergoing ICI treatment.

The 1-year OS was 92% (95% CI: 0.54, 0.99; Supplementary Figure 3). There was no significant difference in OS for patients with and without DMTs (*P* = .16, Supplementary Figure 4). Cox regression did not show any factors significantly related to OS (Table 4). Patients who did not have MS DMT had a nonstatistically significant lower hazard of death (HR = 0.169; 95% CI: 0.002, 15.372; *P* = .4402) compared to patients who had MS treatment.

**Table 4.** The Results from Cox Regression on Overall Survival (OS)

Factor	Univariate analysis	
	HR (95% CI)	*P*-value
Age (per 1-y increase)	0.896 (0.739, 1.088)	.2687
Sex Female vs. male	0.110 (0.001, 10.284)	.3403
Stage II/III vs. IV	0.233 (0.006, 9.746)	.4444
Charlson comorbidity index (per 1-unit increase)	0.919 (0.481, 1.755)	.7981
Smoking Never vs. current/former	1.081 (0.783, 1.494)	.6350
MS Treatment (at ICI initiation) No vs. yes	0.169 (0.002, 15.372)	.4402

Abbreviations: CI, confidence interval; HR, hazard ratio; ICI, immune checkpoint inhibitor; MS, multiple sclerosis.

The median PFS (95% CI) was 7.9 (0.7, N/A; Supplementary Figure 5) When compared between MS patients with and without DMTs, there was no significant difference in PFS (*P* = .2, Supplementary Figure 6). Interestingly, Cox regression analysis showed that the risk of disease progression decreased by 10.3% for every additional year of age (HR = 0.897; 95% CI: 0.823–0.976; *P* = .0120; Table 5). No other factors significantly affected the PFS.

**Table 5.** The Results from Cox Regression on Progression-Free Survival (PFS)

Factor	Univariate analysis	
	HR (95% CI)	*P*-value
Age (per 1-y increase)	**0.897 (0.823, 0.976)**	**.0120**
Sex Female vs. male	1.599 (0.192, 13.341)	.6643
Race Black/AA vs. White	0.746 (0.089, 6.248)	.7866
Stage II/III vs. IV	0.131 (0.015, 1.121)	.0635
Adjuvant No vs. yes	3.192 (0.377, 27.00)	.2866
Provider Academic vs. community	2.623 (0.116, 59.196)	.5442
ECOG performance 0/1 vs. 2	1.499 (0.334, 6.725)	.5970
Charlson comorbidity index (per 1-unit increase)	0.947 (0.746, 1.203)	.6565
Smoking Never vs. current/former	0.533 (0.064, 4.456)	.5612
MS treatment (at ICI initiation) No vs. yes	0.340 (0.047, 2.456)	.2849

Abbreviations: AA, African American; CI, confidence interval; ECOG, Eastern Cooperative Oncology Group; HR, hazard ratio; ICI, immune checkpoint inhibitor; MS, multiple sclerosis.

## Discussion

ICIs have revolutionized cancer therapy and have provided treatment opportunities for previously untreatable cancers. However, they are not typically offered to patients with autoimmune diseases such as MS due to fear of exacerbations and irAEs. This limits the data on the safety and efficacy of ICIs in these patients. In our study of 12 MS patients with solid organ tumors undergoing ICI treatment, we showed that (1) none developed MS relapse or had new brain MRI lesions; (2) 16.7% developed severe irAEs; and (3) the ICI treatment was effective in this cohort with an objective response rate of 50%, 1-year OS of 92%, and median PFS of 7.9 months.

Our study supports prior results that the risk of MS relapse in MS patients treated with ICIs for solid tumors is low.^14–20^ This is interesting given that ICIs enhanced the function of T cells,^2^ which play a central role in the pathogenesis of MS,^25^ and ICIs have been linked to MS flare and *de novo* demyelinating disease.^6^ One potential explanation is that the pathogenesis of ICI-induced demyelination differs from MS exacerbation. This is supported by the fact that there are differences in the molecular and immune responses in arthritis-irAE and classic autoimmune arthritis.^26^ Further studies comparing the pathogenesis of ICI-induced MS flare and classic MS exacerbation are needed to test this possibility.

Compared to prior studies with ICI use in MS patients with cancer,^14–18^ our cohort had a higher mean age (62.2 years), which might represent patients with less active MS because the risk of MS relapse decreases with age.^27,28^ However, the patients included in our studies are more representative of MS patients undergoing solid organ cancer treatment because the most prevalent age group of MS is 55–64 years^29^ and the incidence of cancer increases with age.^30^ The patients included in the study were likely to be in an inactive phase of multiple sclerosis given the long duration of MS diagnosis (median of 22.3 years) and last MS relapse (median of 21.3 years) to ICI treatment. The fact that most patients were not on DMTs and only one-third of the patients were on moderate-efficacy DMTs (dimethyl fumarate, glatiramer acetate, and ainterferon beta-1a)^31^ further supported that the included patients were in the quiescent stage of MS. It is very likely that only patients with relatively inactive MS were considered for ICI treatment by the providers, given the fear of severe relapse in active MS patients.

To our knowledge, there is no study on the interaction between MS DMTs and ICIs. Nonetheless, MS DMTs are sometimes discontinued before the initiation of ICIs due to fear of MS DMTs suppressing immune functions.^32^ On the other hand, cessation of MS DMTs is associated with MS relapses^33^ and prior studies in MS patients undergoing ICIs raised a possibility of MS relapse due to discontinuation of DMTs.^16,17^ In our study, none of the patients with concurrent MS DMT stopped the medication and there were no MS relapses, so we cannot refuse the possibility that MS flare in other studies might be caused by the discontinuation of MS DMTs.^16,17^

Overall, 33.3% of the patients experienced irAE, which is similar to 20–50% found in other studies in the general population and 37% reported in the MS population.^19,34,35^ The severe irAE (grade ≥ 3) found in this study was acute pneumonitis, which was found in 16.7% of the patients. This incidence of acute pneumonitis in our study is higher than 1.3–11% in other studies in general cancer patients.^36,37^ On the other hand, we found that the incidence of low irAE-grade dermatitis in this population was 16.7%, which was lower than that found in other studies.^38^ This means that at least in our study, MS patients receiving ICIs have a comparable incidence of irAEs to the general population; however, whether MS patients undergoing ICI treatment developed a higher incidence of acute pneumonitis would require further study. Interestingly, our study revealed that the risk of severe irAE decreased by 14% for every additional year of age in MS patients. This was in agreement with the decrease in risk of irAE requiring hospitalization with age shown in a study of a large database of more than 14 378 participants,^39^ despite smaller studies showing no significant differences in toxicity with ages.^40–42^ The lowered risk of severe irAE in older patients might in part be explained by immune senescence, more cautious treatments, and different goals of care in the older population.^39^ In summary, ICIs may be safely used in MS patients with a low risk of MS flare and potentially comparable risk of overall irAEs in other studies in the general population.

For the cancer outcomes and efficacy of ICI treatment, our cohort of mostly nonmelanoma solid organ tumor patients (91.7%) and 1 melanoma patient (8.3%) showed an objective response rate of 50% to ICIs. The response rate was higher compared to the rate in the general population of most solid organ tumors (15–30%) but more similar to the response rate in patients with melanoma and MSI-H (45–60%) tumors.^43^ This could be from a greater immune reaction in patients with autoimmunity^44^ or the more stringent selection of patients with underlying MS to receive ICIs compared to the general population due to the perceived increased risk of MS flare and irAE in the former group. There are no studies comparing the objective response rate of MS patients and the general population to our knowledge; however, studies in patients with preexisting autoimmune diseases showed similar or increased efficacy of ICIs when compared to the general population.^44,45^ Thus, ICIs could provide benefits in MS patients with solid organ tumors.

Of note, we found that the risk of disease progression is decreased by 10% for every additional year of age in our population but age was not associated with OS. Although most studies have not seen a difference in PFS between younger and older populations,^46,47^ 1 retrospective study in NSCLC patients found a U-shaped association between age and PFS with the maximum PFS in age of 70–79 years but no difference in OS across most age groups.^48^ This might explain our results, given that the oldest person included in our study was 77.9 years old, which would be within the peak PFS age group from the study. This relationship between older age and decreased risk of disease progression might be from different tumor characteristics, host factors such as immune senescence, and underlying disease state^48^; however, further validation through larger studies is needed to confirm the result.

Interestingly, we found no difference in irAE, PFS, or OS between patients with and without DMTs; however, the nonsignificant trend could suggest lower irAE incidence, risk of disease progression, and hazard of death in the patients without DMTs. This is unexpected because the DMTs used by the included patients (dimethyl fumarate, glatiramer acetate, and interferon beta-1a) all cause an anti-inflammatory immune response. Dimethyl fumarate or Tecfidera works through Nrf-2-dependent and independent pathways, while glatiramer acetate shifts the proinflammatory Th1 T-cells and cytokine (IL-2 and IL-12) to regulatory Th2 T-cells and anti-inflammatory cytokines (IL-1, IL-4, and IL-10).^9^ Interferon beta acts by reducing antigen presentation and T-cell proliferation.^9^ These mechanisms would in theory counteract the effects of T-cell activation and proliferation of ICIs.^2^ These similarities of irAE, PFS, and OS of those with and without DMTs could potentially be because both groups were in the inactive phase of MS; however, the interpretation of the result might also be limited by the small number of patients and the inability to perform multivariate analysis and further larger studies are warranted to clarify the relationship. Given no differences in irAE, PFS, and OS in MS patients with and without DMTs, continuation of DMTs during ICI treatment might be reasonable, given the known risk of MS relapse after cessation of DMTs.^33^

Our study has several strengths. We are the first to report and analyze the efficacy (in terms of objective response rate, OS, and PFS) of ICIs in patients with MS, and show that ICIs are effective in treating cancer in these patients. We explored the factors affecting the safety and efficacy of ICI use and found that age decreases the risk of irAEs. We also pioneered the comparison between MS patients with and without DMTs in safety and efficacy outcomes, showing that concurrent use of moderate-efficacy DMTs does not affect the risk of the irAEs or efficacy of ICIs in MS patients.

The main limitations of this study include a small sample size, which prevented further evaluation with multivariate analysis and multiple comparisons due to insufficient power. The results from univariate analyses can suggest trends; however, they should be interpreted with caution of possible false positives. The small sample size with heterogeneity in oncologic diseases and immune checkpoint inhibitors also precluded analysis of specific cancer types or stages. Secondly, the study has limited generalizability. The patients included were mostly in an inactive phase of MS with advanced age and were not on high-efficacy DMTs, so study results might not apply to broader MS patients who have active disease or are on high-efficacy DMTs. Given that T cells are central to the function of ICI^2^ and the pathogenesis of MS,^6,7^ patients with active MS might experience more MS relapse and irAEs with ICI use. Moreover, the studied patients were predominately white, therefore the results might not apply to MS patients from other races. Other limitations are from the retrospective nature of the study, which includes missing data and lost follow-up,^49^ and a relatively short follow-up time (median of 14.7 months). Larger longitudinal studies and/or meta-analyses are needed to confirm the tolerability and efficacy of ICIs in MS patients in comparison to the general population, clarify if age affects the irAE risk and PFS, and investigate the outcomes of ICI treatment in MS patients with and without DMTs. The inclusion of more active MS patients in studies would also expand the generalizability to broader MS patients.

In conclusion, our study of 12 MS patients suggests that ICIs might be safely used with a low risk of MS flare and comparable irAE risk with the general population in selected patients with inactive MS. ICIs could potentially be used effectively in this group of patients and MS patients, especially those in an inactive phase of MS, should not be excluded from consideration of ICI treatment. This data also suggests that MS DMTs could be continued during ICI treatment, however, larger studies or meta-analyses are required to confirm these data.

## Supplementary Material

vdaf048_suppl_Supplementary_Figures

## Data Availability

Data and materials are available from the corresponding author upon reasonable request.
