# Supplementary material for: Outcomes in patients with multiple sclerosis and solid organ cancers treated with immune checkpoint inhibitors
Source: Neurooncol Adv. 2025 Mar 4;7(1):vdaf048. doi: 10.1093/noajnl/vdaf048 (PMC12012678; doi:10.1093/noajnl/vdaf048)
Supplement: vdaf048_suppl_Supplementary_Figures [file vdaf048_suppl_supplementary_figures.docx]

## Supplementary Figures


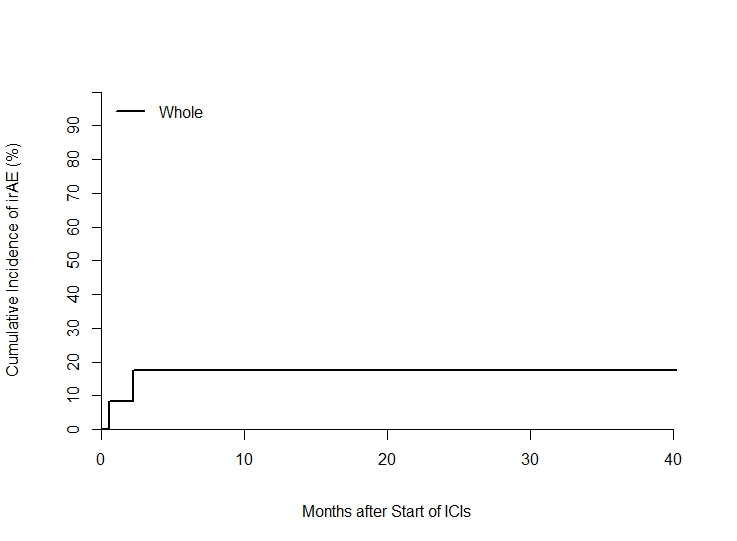


**Supplementary Figure 1.** Cumulative incidence of having severe irAE of the whole cohort

Abbreviations: ICI, immune checkpoint inhibitor; irAE, Immune-related adverse


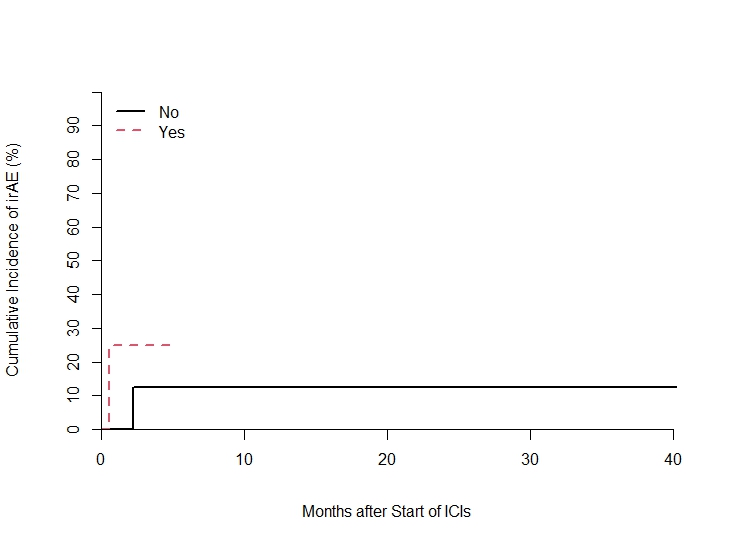


**Supplementary Figure 2.** Cumulative incidence of having severe irAE by MS treatment. The red line represents MS patients with concurrent DMTs. The black line represents MS patients without concurrent DMTs. There was not a significant difference (p=0.3908).

Abbreviations: DMT, Disease-modifying therapy; ICI, immune checkpoint inhibitor; irAE, Immune-related adverse; MS, Multiple sclerosis


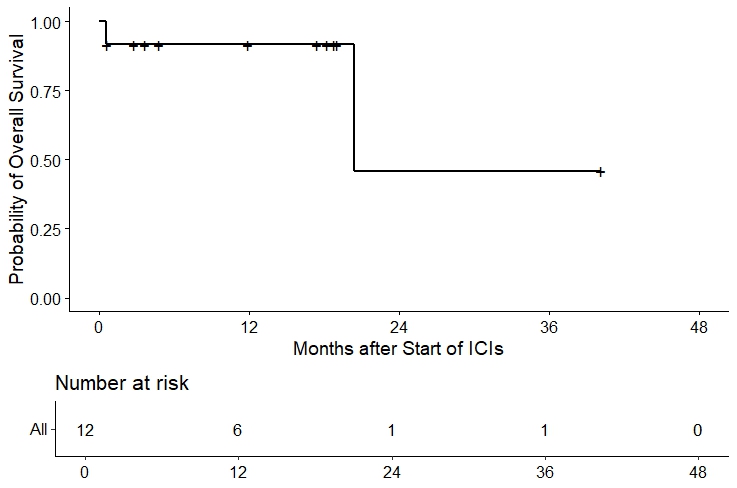


**Supplementary Figure 3.** Kaplan-Meier plot of OS for the whole cohort

Abbreviations: ICI, immune checkpoint inhibitor; OS, overall survival


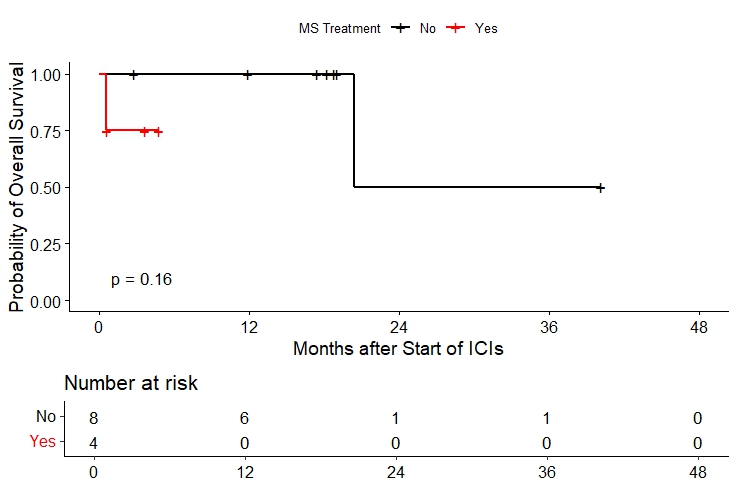


**Supplementary Figure 4.** Kaplan-Meier plot of overall survival (OS) by MS treatment. The red line represents MS patients with concurrent DMTs. The black line represents MS patients without concurrent DMTs. There was no significant difference in OS (p=0.16).

Abbreviations: DMT, Disease-modifying therapy; ICI, immune checkpoint inhibitor; MS, Multiple sclerosis; OS, overall survival


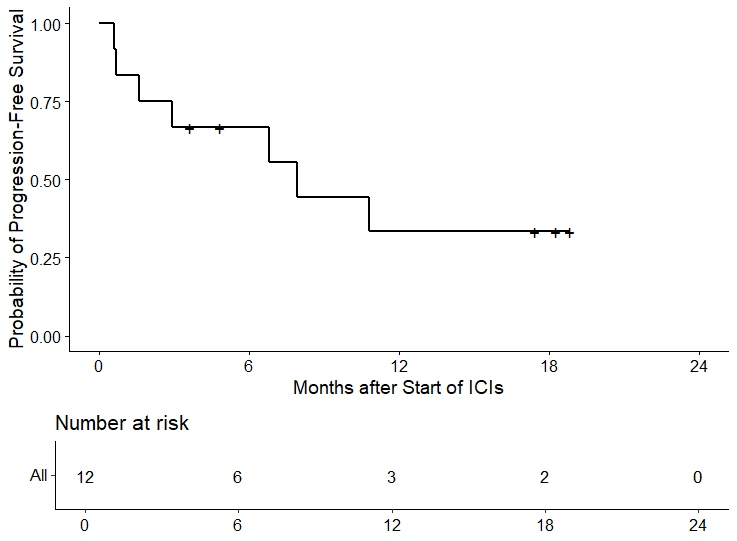


**Supplementary Figure 5.** Kaplan-Meier estimation of progression-free survival (PFS) of the whole cohort.

Abbreviations: ICI, immune checkpoint inhibitor; PFS, progression-free survival


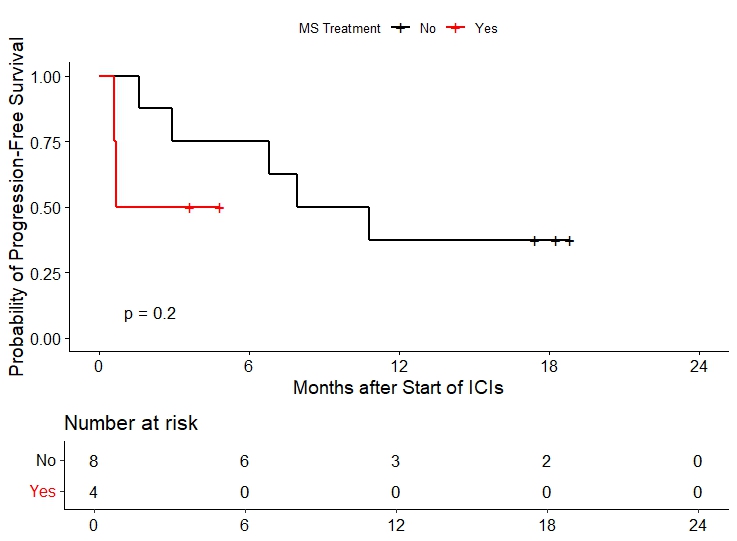


**Supplementary Figure 6.** Kaplan-Meier estimation of progression-free survival (PFS) by MS treatment. The red line represents MS patients with concurrent DMTs. The black line represents MS patients without concurrent DMTs. There was no significant difference in PFS (p=0.2).

Abbreviations: DMT, Disease-modifying therapy; ICI, immune checkpoint inhibitor; MS, Multiple sclerosis; PFS, Progression-free survival
